# Platelet, a key regulator of innate and adaptive immunity

**DOI:** 10.3389/fmed.2023.1074878

**Published:** 2023-03-10

**Authors:** Cheng Yan, Haojie Wu, Xianchun Fang, Junji He, Feng Zhu

**Affiliations:** ^1^Department of Blood Transfusion, Nanjing Jiangning Hospital, The Affiliated Jiangning Hospital of Nanjing Medical University, Nanjing, Jiangsu, China; ^2^Department of Laboratory Medicine, The First Affiliated Hospital of Nanjing Medical University, Nanjing, Jiangsu, China

**Keywords:** platelet, monocyte, macrophage, T cell, B cell, dendritic cell

## Abstract

Platelets, anucleate blood components, represent the major cell type involved in the regulation of hemostasis and thrombosis. In addition to performing haemostatic roles, platelets can influence both innate and adaptive immune responses. In this review, we summarize the development of platelets and their functions in hemostasis. We also discuss the interactions between platelet products and innate or adaptive immune cells, including neutrophils, monocytes, macrophages, T cells, B cells and dendritic cells. Activated platelets and released molecules regulate the differentiation and function of these cells *via* platelet-derived receptors or secreting molecules. Platelets have dual effects on nearly all immune cells. Understanding the exact mechanisms underlying these effects will enable further application of platelet transfusion.

## Introduction to platelets

The production of platelets from megakaryocytes (MKs) is a systematic process that is thought to occur in the bone marrow (1). Thrombopoiesis occurs from common myeloid progenitor (CMP) cells in the bone marrow, which differentiate into promegakaryocytes and then into MKs. After migrating into the vascular niche, mature MKs extend many proplatelets (PPTs) through the sinusoid vessel barrier (2–4). Then, PPTs interconvert into pre-platelets, and platelets are created after the fission of pre-platelets (5). Each MK can produce 1,000–3,000 platelets after multiple divisions (6, 7). A recent study proposed the lung as the main site of platelet release (8). The average lifespan of platelets is only 8–10 days. In the circulation, each individual has 150–400 × 10^9^ platelets per liter of peripheral blood (9).

The primary roles of platelets are hemostasis and thrombosis. Hemostasis is the process that stops blood loss from a damaged vessel (10). Hemostasis involves multiple interlinked steps: primary hemostasis, secondary hemostasis, and tertiary hemostasis (11). Platelets are mainly involved in primary hemostasis, which is also called platelet clotting. In primary hemostasis, platelets stick to the damaged tissue and become activated, which recruits more platelets to form a platelet “plug” to stop blood loss from the damaged area. Primary hemostasis may also involve constriction of the blood vessel, which can occur due to substances released by platelets (12). In addition to hemostasis, platelet activation also contributes to thrombosis, which is a blood clot within a blood vessel that limits the flow of blood. Platelets play a significant role in the development of arterial thrombosis rather than venous thrombosis (13). Atherosclerosis allows the activation of platelets, causing adhesion and aggregation, which leads to the formation of a clot. Thus, the management of arterial thrombosis predominantly involves the use of antiplatelet agents for monotherapy or dual-antiplatelet therapy (14).

For long, Platelets are small, anucleate cell debris (15). Actually, platelets possess almost every feature of cells, except the nucleus. The role of platelets in hemostasis has long been known, but they have also been shown to be involved in defense against pathogens (16, 17), as well as in the acceleration of autoimmune diseases (18). Therefore, platelets are seen as a cellular component of the innate immune system (19). In the presence of certain infectious agents or inflammatory stimuli, platelets mediate hemostasis and thrombosis and activate innate and adaptive immunity *via* specific receptors (CD42, CD41 CD40, CD154, etc.) and/or granule release (CXCL4, CCL5, TGFβ, serotonin, β-defensin, etc.), RNA transfer, and mitochondrial secretion (20). Moreover, it was found that platelets can also release extracellular vesicles (EVs), including ectosomes (also called microvesicles or microparticles; 100–1,000 nm) and exosomes (40–100 nm) to regulate hemostasis, thrombosis and inflammation (21). In this review, we will summarize platelet-mediated regulation of innate and adaptive immune cells.

## Influence of platelets on immune cells

Due to the haemostatic function of platelets, platelet transfusion is used to treat thrombocytopenia platelet function defecting disease in the clinic. Initially, platelet transfusions were thought to have no side effects, but recent findings have indicated that although the effects are not fatal, platelet transfusion can lead to febrile nonhemolytic transfusion reactions (FNHTRs), anaphylactic reactions, haemolytic transfusion reactions and other immune-mediated reactions (22). Guo et al. found that antibody-mediated immune thrombocytopenia (ITP) was resistant to allogeneic platelet transfusions, while the T-cell-mediated form of the disease was susceptible, suggesting that transfusion therapy might be beneficial for antibody-negative ITP (23). Moreover, it was reported that fresh platelets could induce transfusion-related immunomodulation (TRIM) independent of white cells (WBCs) due to their MHC antigen expression, whereas aging results in the loss of MHC and the ability to mediate TRIM (24). Ultraviolet B (UVB) radiation plus riboflavin treatment of WBC-enriched platelet-rich plasma (PRP) effectively blocks alloimmunization and modulates immune responses to subsequent exposures (25). These reports demonstrated that various reactions mediated by different WBCs led to limitations in the application of platelet transfusion. We detail the interactions between platelets and different WBCs below.

## Neutrophils

As an indispensable member of the innate immune system, neutrophils are the first leukocytes to infiltrate the site of injury (26). Platelet derived P-selectin induces neutrophils to move to sites of thrombus formation by activating P-selectin glycoprotein ligand-1 (PSGL1), a receptor of P-selectin on neutrophils (27). The platelet-derived serotonin metabolite 5-hydroxyindoleacetic acid (5-HIAA) also promotes neutrophil recruitment to inflamed tissue *via* the G-protein-coupled receptor 35 (GPR35) (28). In a murine model of *Klebsiella pneumoniae*-induced pulmonary inflammation, Toll-like receptor 4 (TLR4) deficiency in platelets decreased the number of neutrophils in the lung (29). Inhibition of platelet p110β (the catalytic subunit of phosphatidylinositol 3-kinase) prevented platelet–neutrophil interactions, diminishing neutrophil infiltration (30). It was also reported that activated platelet-derived nanovesicles could recruit neutrophils to exert anti-tumor effects (31). C-type lectin-like receptor (CLEC-2) was recently discovered as a platelet receptor. Blocking platelet CLEC-2 signaling enhanced liver recovery from acute toxic liver injuries by increasing tumor necrosis factor-α (TNF-α) production, which then improved reparative hepatic neutrophil recruitment (32). All these findings indicated that platelets regulate the movement of neutrophils.

Platelets also regulate neutrophil activation. Mac1 and LFA1 strengthen the attachment between platelets and neutrophils *via* junctional adhesion molecule 3 (JAM3) (33), Intercellular adhesion molecule 2 (ICAM-2) (34), CD42 (35) and Choline transporter-like protein 2 (CTL2) (36), which bind to platelet ɑIIbβ3 integrin, enhancing neutrophil activation. Clinical observational data showed that the levels of C-X-C chemokine receptor type 4 (CXCL4; also called platelet factor 4, PF4), CXCL7 (neutrophil activating protein-2, NAP2) and myeloperoxidase (MPO) were related to platelet activation and platelet–neutrophil interactions (37). Both CXCL4 and CXCL7 secreted by platelets can initiate neutrophil activation (38, 39). Similarly, inhibition of the chemokine receptors CXCR4 and CXCR7 on platelets and polymorphonuclear neutrophils (PMNs) was shown to reduce platelet–neutrophil complex (PNC) formation (40). Leukotriene B4 (LTB4) and leukotriene A4 (LTA4) derived from platelet-derived arachidonic acid (AA) can activate neutrophils (41). In addition, platelet-derived mitochondria induce the release of human neutrophil microvesicles that recruit additional immune cells to remove pathogens (42). Platelet-derived serotonin was shown to promote neutrophil degranulation, which increased the expression of the membrane-bound leukocyte adhesion molecule CD11b, enhanced inflammation in the infarct area and reduced myocardial salvage by inducing the release of myeloperoxidase and hydrogen peroxide (H_2_O_2_) (43).

An important function of neutrophils is to release neutrophil extracellular traps (NETs), which remove pathogens from the circulation. Platelet-derived exosomal high-mobility group protein 1 (HMGB1) and/or miR-15b-5p and miR-378a-3p promote excessive NET formation through the Akt/mTOR autophagy pathway during sepsis and subsequent organ injury (44). It was reported that both HMGB1 and the C3a component released by platelets could activate neutrophils to induce the formation of NETs (45–47). These stimuli significantly enhanced PSGL-1-induced neutrophil activation. Additionally, platelets interact with C3b attached to NETs (48). The P-selectin-PSGL-1 interaction was shown to induce the release of NETs, and clearing activated platelets *via* platelet-derived microparticle (100–1,000 nm) mediated neutrophil activation (49). Moreover, platelets were able to induce NOX-independent NET formation in a dengue virus non-structural protein 1 (NS1)-dependent manner (50). The inhibition of nuclear factor of activated T cells (NFAT) in platelets promotes interactions with neutrophils and NET induction, which might be harnessed in the clinic (51). In addition to influencing the infiltration of neutrophils, the TLR4-ERK5 axis in platelets facilitates NET formation to promote the capture of circulating tumor cells (52). Furthermore, a neutralizing anti-CXCL4 antibody significantly inhibited NET formation induced by NCA-associated vasculitis (AAV)-derived platelets (38).

Interestingly, NETs also induce a hypercoagulable state in platelets by upregulating phosphatidylserine and P-selectin on cells in the context of gastric cancer (GC) (53). Neutrophils can activate platelets by releasing antimicrobial cathelicidins *via* degranulation or as part of NETs. For example, cathelicidin LL-37 and its mouse homolog cathelicidin-related antimicrobial peptide (CRAMP) can bind glycoprotein VI (GPVI) on the platelet surface, further stimulating platelet and neutrophil activation (54). Citrullinated H3 histones, key markers of ongoing NETosis, have also been shown to activate platelets (48). During ischaemic stroke, neutrophils can rapidly bind platelets through P-selectin and glycoprotein Ibα (55), and neutrophils have been shown to undergo “plucking” on megakaryocytes to accelerate platelet production *via* CXCR4-CXCL12 signaling (56). Additionally, neutrophils activate platelets after pneumolysin exposure by releasing extracellular vesicles (EVs; 100–1,000 nm) (57). In acute myocardial infarction, the observed increase in S100A8/A9 levels in platelets was not due to an increase in synthesis but was due to uptake of proteins secreted by neutrophils (58). This result indicated that neutrophils were able to alter the platelet proteome.

Taken together, these findings support mutual regulation between platelets and neutrophils. Platelets can regulate infiltration, activation and NET formation in neutrophils (Table 1). In the clinic, depending on the specific conditions of a disease, the mechanism of mutual regulation between these cell types could be controlled or blocked.

**Table 1:** Platelet-derived molecules affect the function of neutrophils.

Regulating aspects on neutrophils	Platelet components	Regulatory effect	References
Infiltration	P-selectin, 5-HIAA, p110β	Upregulated	(25, 26, 28)
	TLR4, CLEC-2	Downregulated	(27, 30)
Activation	ɑIIbβ3, CXCL4, CXCL7, MPO, LTB4, LTA4, mitochondria, serotonin	Upregulated	(35–37, 39–41)
NET formation	HMGB1, miR-15b-5p, miR-378a-3p, C3a, C3b, P-selectin, TLR4, CXCL4, NS1	Upregulated	(42–48)
	Nuclear factor of activated T cells (NFAT)	Downregulated	(49)

## Monocytes

Platelets mediate multiple types of immune responses, and many studies have shown that platelets can interact with innate immune cells during infection and inflammation. One study showed that platelet activation is the major initiator of platelet–monocyte aggregation (59). Platelets interact with monocytes through cluster of differentiation (CD)62p (known as P-selectin), which recognizes PSGL-1 expressed on the surface of monocytes to initiate aggregation (60). Platelet-derived hyaluronidase-2 (HYAL2) also causes aggregate formation (61). Platelets from severe COVID-19 patients were highly activated and induced the expression of tissue factor (TF) in monocytes from healthy volunteers (62). The increased expression of TF also drives platelet–monocyte aggregation (63), inflammatory activation and inflammatory cytokine secretion (64). However, activated platelet-derived EVs (40–100 nm) contribute to the suppression of TF expression by transferring hsa-miR-223-3p to monocytes, which inhibits aggregation (65). Therefore, the exact influence of platelets on platelet–monocyte aggregation needs to be further explored.

Platelets can induce the oxidative burst and inflammation in monocytes and neutrophils *via* direct interactions (66–70) and promote leukocyte adhesion and extravasation (71–73). In addition, platelet aggregability leads to monocyte extravasation into the infarcted myocardium and influences inflammation in patients with acute myocardial infarction (74). The SARS-CoV-2 spike protein can interact with the CD42b receptor to activate platelets and promote proinflammatory cytokine production by monocytes through the interaction of P-selectin/PGSL-1 and CD40L/CD40 (75). P-selectin was shown to contribute to the secretion of TNFα, IL-1β, IL-6, CXCL8, IL12 and CCL4 by autologous monocytes (76, 77). In addition, platelets were reported to potentiate the release of IL-8, mainly from monocytes (78). Thus, some researchers have concluded that platelet–monocyte aggregates can be used as a robust marker of platelet activation and monocyte inflammatory responses (79). Interestingly, platelets do not always activate monocytes to induce the production of proinflammatory cytokines, and platelet-monocyte interactions can actually decrease inflammation by increasing IL-10 levels and reducing TNF-α levels in monocytes through CD40L/CD40 (80).

Platelets also regulate the differentiation of monocytes. Both Sigrun Badrnya et al. and J H Phillips et al. reported that activated platelets increased CD16 expression, which induced monocytes to switch to an intermediate phenotype. CD16^+^ monocytes produced transcripts for the gamma subunit of the high-affinity IgE FcR and could kill anti-CD16 hybridoma cell targets in the absence of CD3 zeta (81, 82). Similarly, it was reported that platelet-derived CXCL4 induced monocyte differentiation into macrophages (83). However, another study reported that inhibition of PSGL-1 or P-selectin did not attenuate platelet-mediated monocyte activation (84). This meant that there were other pathways activating monocytes. Zachary et al. found that platelet-derived β-2 microglobulin (β2M) induced monocyte proinflammatory differentiation through a noncanonical TGFβ receptor pathway (85) and regulated age-associated monocyte polarization. β2M was shown to maintain the balance between inflammatory and reparative signals. In addition, loss of β2M increases profibrotic cardiac responses (86). Thus, platelets have both pro- and anti-inflammatory effects on monocytes (Table 2).

**Table 2:** Platelet-derived molecules affect the function and differentiation of monocytes.

Platelets products	Receptors on monocytes	Effects on monocyte function	References
CD40L	CD40	IL-10 ↑, TNF-α ↓	(39)
P-selectin	PSGL-1	TNFα, IL-1β, IL-6,IL-8 CXCL8, IL-12, CCL4↑	(19, 35, 36)
Hyaluronidase-2 (HYAL2)	/	Platelet–monocyte aggregation ↑	(20)
Extracellular vesicles (EVs)	Tissue factor	Platelet–monocyte aggregation ↑	(24)
Platelet factor 4 (PF4)	/	Differentiating into macrophages ↑	(42)
β-2 micro globulin (β2M)	Noncanonical TGFβ receptor pathway	Proinflammatory differentiation ↑	(44)

## Macrophages

The phenotype and function of macrophages are also affected by platelets. Haem-activated platelets promote the formation of macrophage extracellular traps (METs) *via* reactive oxygen species generation or histone citrullination, enhancing rhabdomyolysis-induced acute kidney injury (87). Platelet-conditioned medium was also shown to induce an anti-inflammatory, pro-resolving phenotype in macrophages (88). Platelet-Treg cell aggregates in the lung induce macrophage polarization toward an anti-inflammatory phenotype and promote effective resolution of pulmonary inflammation (89). Ryoka et al. reported that platelet-rich plasma (PRP) suppressed M1 macrophage polarization and promoted M2 macrophage polarization (90). Platelet-rich fibrin could shift macrophage polarization from an M1 phenotype toward an M2 phenotype to induce an anti-inflammatory response (91) and reduce IL-1β release and caspase-1 production in macrophages that underwent pyroptosis by increasing NLR family pyrin domain containing 3 (NLRP3) ubiquitination (92, 93). Interestingly, both leukocyte-poor (LP) and leukocyte-rich (LR) PRP promoted the recruitment of M1 macrophages during the process of tendon healing, while the number of M2 macrophages was high only in the LP-PRP group (94). However, the presence of platelets skewed monocytes toward an M1 phenotype *via* the GPIb-CD11b axis in the presence of lipopolysaccharide (LPS) (95). Thus, platelets can affect macrophage polarization *via* different pathways.

## Natural killer cells

Natural killer cells are also regulated by platelets. PLT-ectosomes (100–1,000 nm) can inhibit NK cell effector function in a TGF-β1-dependent manner, reducing the expression of surface receptors, like natural-killer group 2, member D (NKG2D), natural-killer p30 (NKp30) and CD226, and IFN-γ production (96). This result suggested that platelets can promote tumor dissemination by coating tumor cells (97, 98). Co-incubation of NK cells with platelets was shown to reduce NK cell cytotoxicity by reducing NK cell degranulation, IFN-γ production, NKG2D and natural-killer p46 (NKp46) expression and increased Killer cell immunoglobulin-like receptor 2DL1 (KIR2DL1) expression in NK cells (99). Furthermore, NK cell cytolytic activity was shown to be attenuated *via* tumor cell-induced platelet secretion (100). However, platelet-derived growth factor D (PDGF-DD)-activated IL-2-induced NK cells exert anti-tumor effects by binding with the NKp44 receptor (101), and PDGF-D-PDGFRβ signaling enhances IL-15-mediated human NK cell survival (102). In addition, platelet-derived CXCL4 induces human natural killer cells to synthesize and release interleukin-8 (103). Taken together, these findings show that platelets also have dual regulatory effects on NK cells.

## T cells

In addition to innate immune cells, platelets also influence T-cell and B-cell responses. Norbert et al. found that platelets enhanced the differentiation and cytokine production of CD4^+^ T cells *via* both direct cell–cell contact and multiple chemokines (platelet-derived CXCL4 and CCL5) (104). Platelets can produce many molecules, such as FasL, TNF-related apoptosis-inducing ligand (TRAIL), IL-7 and CD40L, which are all important for adaptive immune responses (105–107). In particular, platelet CD40L regulates CD8^+^ T-cell response. Elzey et al. reported that depletion of platelets decreased the generation of cytotoxic T lymphocytes (CTLs) (108). In platelet-depleted mice, reconstitution of platelets increased the number of CTLs in the spleen and liver after lymphocytic choriomeningitis virus (LCMV) infection (109). Thus, platelets are important for the expansion of antigen-specific CTLs. Chapman et al. also demonstrated that platelets can process and present antigens *via* MHC class I and directly activate naive T cells in a platelet MHC class I-dependent manner (110). However, platelets were also shown to delay the infiltration of CD8^+^ T cells into the liver, allowing increased viral replication *via* the release of serotonin, which might ultimately cause chronic hepatitis (111). In addition, Aslam et al. found that platelets suppressed CD8^+^ T-cell function in a transfusion-related model and that transfusion of MHC-I bearing platelets prolonged allograft survival (24). Interestingly, the expression of MHC-I in platelets was shown to be significantly increased in humans and mice, which reduced the numbers and impaired the function of antigen-specific CD8^+^ T cells during sepsis (112). Platelets promote protection against *C. albicans* airway mycosis by activating Th2 and Th17 responses *via* an antifungal pathway that includes candidalysin, GP1bα, and dickkopf WNT signaling pathway inhibitor 1 (Dkk-1) (113). However, activated platelets accumulate in the lung lesions of tuberculosis patients and inhibit T-cell responses and *mycobacterium tuberculosis* replication in macrophages (114). CD84-lacking platelets were shown to reduce cerebral CD4^+^ T-cell infiltration and thrombotic activity, slowing neurological damage in an experimental model of stroke. In a clinical study, a high level of platelet CD84 expression resulted in poor outcomes in patients with stroke (115). Platelets block the immunosuppressive function of Tregs directly *via* the P-selectin/PSGL-1 axis, which induces Syk phosphorylation and an increase in intracellular calcium in systemic lupus erythematosus (SLE) patients (116). However, Jan et al. reported that interactions between platelets and Tregs *via* the P-selectin/PSGL-1 axis encouraged the release of the anti-inflammatory mediators IL-10 and TGFβ. Platelet-Treg cell aggregates induce macrophage polarization toward an anti-inflammatory phenotype in pulmonary inflammation (89). Rachidi et al. found that platelets constrained T-cell immunity through a glycoprotein repetition predominant (GARP)-TGFβ axis, and platelet-specific deletion of GARP potentiated protective immunity against both melanoma and colon cancer (117). In addition, Hinterleitner et al. reported that platelet-derived GARP induced peripheral Treg cells by upregulating Foxp3 expression (118).

Platelet-derived CXCL4 was also shown to enhance Th1 cell responses and CD4^+^ T effector memory cell responses *via* Akt-PGC1α-TFAM signaling-mediated mitochondrial biogenesis (119). Platelets exert dose-dependent regulatory effects on the effector responses of naive T cells *via* CXCL4-TGFβ. Low concentrations of CXCL4 reinforce TGFβ signaling, but high concentrations of CXCL4 have the opposite effect (120). In addition, knocking down the expression of CXCR3, the receptor of CXCL4, was shown to abolish Th1 and Treg cell responses (121). Platelet-derived mitochondria directly upregulate central memory (TCM) CD4^+^ T cells and downregulate effector memory (TEM) CD4^+^ T cells through C-X-C motif chemokine receptor 4 (CXCR4) and its ligand stromal cell-derived factor-1 (SDF-1) (122). However, CXCL4 expression is inversely correlated with T-cell function in advanced lung adenocarcinoma (LAC), leading to accelerated development of tumors (123). Pten-deficient platelets are hyperactive and overproduce multiple Tfh-promoting cytokines *via* the PDK1/mTORC2-AKT-SNAP23 axis, which promotes CD4^+^ T-cell differentiation into Tfh cells. Pten deletion results in age-related lymphoproliferative diseases and humoral autoimmunity in mice (124).

**Figure 1:**
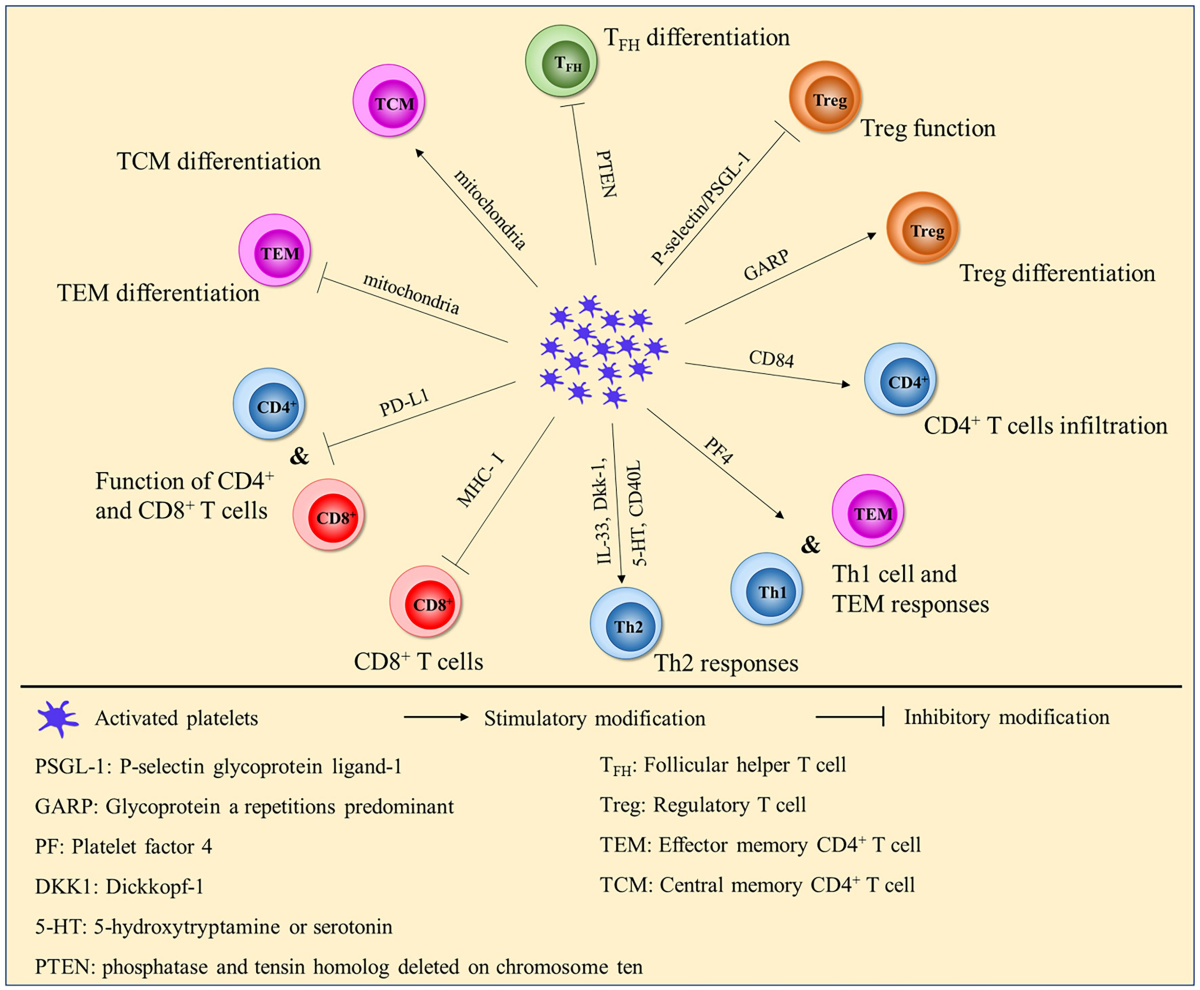
Platelets affect the function and differentiation of CD4^+^ and CD8^+^ T cells.

PD-L1 is widely known as an inhibitor of the adaptive immune system. It can be transferred from tumor cells to platelets in a fibronectin 1-, integrin α5β1- and GPIbα-dependent manner in non-small cell lung cancer (NSCLC), and platelet PD-L1 possesses the ability to inhibit the function of CD4^+^ and CD8^+^ T cells (118). Christina et al. demonstrated that platelets decrease PD-1 and PD-L1 expression, T-cell proliferation and IFN-γ and TNF-α production (125). PD-L1-overexpressing platelets can rescue β-cells by suppressing the activity of pancreatic autoreactive T cells and increasing the percentage of Tregs in type 1 diabetes (T1D) (126). However, high expression of PD-L1 was found in platelets from COVID-19 patients, which inhibited the upregulation of CD25 expression and TNF-α and IFN-γ production by CD4^+^ T cells (127). Activated platelet-derived IL-33, Dkk-1, and 5-HT or CD40L induce type 2 immune response or interact with TSLP-stimulated myeloid DCs to tune the sensitization stage of allergic asthma through RANK/RANKL signaling (128). In addition, platelet-CD4^+^ T-cell aggregate frequency was positively correlated with HIV-1 viral load and was related to immune activation during HIV-1 infection (129). HIV-containing platelets result in dysfunction of glycolysis-mediated energy production in CD4^+^ T cells. This result indicates that platelets might be a therapeutic target for immunological non-responders (130) (Figure 1).

## B cells

Compared with those on T cells, the effects of platelets on B cells are less well studied. It was reported that the transfer of normal platelets into CD40L-deficient mice could transiently increase antigen-specific IgG production (131, 132). Fabrice et al. also reported that platelets could activate peripheral blood B cells and increase the production of immunoglobulins (133). These results indicated that platelet CD40L contributed to B-cell responses when CD4^+^ T-cell-derived CD40L was absent. CD40L released from platelets contributes to the proliferation of tumor cells in intravascular large B-cell lymphoma (134). In addition, platelets secreting PF4 increase B-cell differentiation in the bone marrow environment by inducing the phosphorylation of STAT5 (135).

Since the start of the COVID-19 pandemic, vaccines for SARS-CoV-2 have been developed. In addition, numerous researchers have found that vaccine-induced immune thrombotic thrombocytopenia (VITT) occurs in individuals exposed to vaccines, especially adenoviral vector vaccines (136). VITT is an autoimmune condition characterized by antibodies that directly activate platelets, triggering thrombosis in the arterial and venous circulation. The pathophysiology of VITT is still incompletely understood (137). One hypothesis suggested that vaccines might bind to PF4, creating a novel antigen that is subsequently taken up by monocytes and trafficked to the lymph nodes, where it stimulates the proliferation of anti-PF4 memory B cells. Then, antibody binding to FcγRIII-A contributes to platelet clearance and thrombocytopenia (138). Additionally, platelets express human Fc receptors. The receptors for IgG, the Fc-γ receptor, and IgE, the Fc-ε receptor, are expressed on the platelet surface (139). FcγRIIA-expressing platelets activated by IgG immune complexes contribute to the severity of anaphylaxis (140). IgE antibody binding to platelets *via* the low-affinity IgE receptor (Fc epsilon RII/CD23) or high-affinity IgE receptor (Fc epsilon RI) led to immediate-type allergic reactions (141, 142). Thus, platelets and their secreted molecules can influence B cells in adaptive immunity. In addition, antibodies released by B cells are also able to regulate the numbers and activation of platelets.

## Dendritic cells

Although many studies have indicated that platelets play critical roles in T-cell and B-cell adaptive immunity, the mechanism is still unknown. In addition to their ability to mediate T-cell and B-cell immune responses directly, platelets might also regulate these responses indirectly through dendritic cells (DCs). DCs are the primary antigen-presenting cells and can cross-present antigens to T cells to induce antigen-specific cell responses. Thus, changes in the number or phenotype of DCs influence cell immunity.

Platelets were reported to significantly inhibit proinflammatory (IL-12, IL-6, and TNFα) cytokine production and increase anti-inflammatory (IL-10) cytokine production in moDCs. In addition, platelet-derived soluble mediators inhibit T-cell priming and T helper differentiation toward an IFNγ^+^ Th1 phenotype induced by moDCs (143). Platelet concentrates also downregulate the expression of CD40, CD80, CD83, and CD86 and IL-8, IL-12 and IL-6 secretion by BDCA^+^ DCs (144, 145). Similarly, platelet-monocyte complex (PMC)-derived DCs were shown to exhibit reduced levels of critical molecules for DC function (CD206, CD80, CD86, and CCR7) and reduced antigen uptake capacity (146). Conversely, thrombin-activated platelets increased CD80 expression in DCs and induced DCs to produce tumor necrosis factor alpha (TNF-α), interleukin 12 (IL-12), and IL-6 after coculture of BMDCs and *staphylococcus aureus in vitro* (147). Furthermore, platelets have the ability to enhance the DC-mediated Th2 response and contribute to allergic inflammation through the RANK ligand (148). Platelets are necessary for efficient host sensitization to allergens and increase the allergen sensitization of CD11c^+^ DCs (149).

Platelet-derived P-selectin interacts with PSGL-1 on the surface of monocytes and induces monocyte differentiation into DCs, which are more potent than cytokine-derived DCs during tumor-specific T-cell immune responses (150). Additionally, the interaction between platelets and DCs is mediated by Mac-1, which is upregulated on DCs by activated platelets in a PSGL-1-dependent manner (151). Moreover, platelet-derived CD40L can induce monocyte differentiation into DCs, promote DC maturation, increase the expression of costimulatory molecules (152, 153) and enhance interferon-α secretion by plasmacytoid dendritic cells in systemic lupus erythematosus (154). Sharmeen et al. also reported that platelets enhanced dendritic cell responses through CD40-CD40L during *staphylococcus aureus* infection (147).

Serotonin, another platelet molecule, had opposite effects on DC differentiation. As the expression of costimulatory molecules on DCs was reduced and IL-10 production was increased by serotonin, the antigen presentation function of DCs was repressed (155). Similarly, CXCL4 inhibited monocyte differentiation into CD1α^+^ DCs and increased the number of CD1α^−^ DCs, but CD1α^−^ DCs were not as effective as CD1α^+^ DCs in activating T cells (156). Moreover, CXCL4 enhanced monocyte-derived DCs to promote autologous CD4^+^ T-cell and CD8^+^ T-cell proliferation and the production of IFN-γ and IL-4 (157). In summary, platelets and their secreted molecules have different effects on the development or differentiation of DCs, and the exact mechanism still needs further exploration (Table 3).

**Table 3:** Platelet-derived molecules affect the function and differentiation of dendritic cells.

Platelet products	Subtype of dendritic cells	Effects on dendritic cells	References
Secretome	mo-DCs	IL-12, IL-6, TNFα, CD206, CD80, CD86, CCR7↓; IL-10↑	(92, 95)
Platelet concentrates	BDCA^+^DCs	CD206, CD80, CD86, CCR7, IL-8, IL-12 and IL-6 ↓	(93, 94)
Thrombin-activated platelet	BMDCs	CD80, TNF-α, IL-12, IL-6↑	(95)
RANK ligand	myeloid DCs	CD86, CD40, CD83, Th2 response↑	(97)
Platelets	CD11c^+^ DCs	allergen sensitization↑	(98)
P-selectin	mo-DCs	Mac-1 expression↑	(100)
CD40L	mo-DCs	Maturation, costimulatory molecules, IL-12p40↑	(101, 102)
CD40L	pDCs	IFN-α↑	(103)
Serotonin	mo-DCs	costimulatory molecules↓; IL-10↑	(105)
CXCL4 (PF4)	mo-DCs	CD1α^+^DCs↓; CD1α^−^DCs↑; IFN-γ and IL-4↑	(106, 107)

## Conclusion

In addition to supporting thrombosis, platelets release a number of mediators that regulate both innate and adaptive immunity. Due to the large number of platelets in the circulation, they and their products can efficiently interact with peripheral circulating cells directly, such as neutrophils, monocytes, T cells, B cells, DCs, macrophages, and NK cells, which modulates their differentiation. Platelets affect the functions of monocytes and neutrophils, including their receptors and soluble mediators. Moreover, platelet interactions with monocytes induce their differentiation into macrophages and regulate cytokine release. Depending on the severity of the disease, platelets can enhance or reduce leukocyte cytokine production, which maintains a balance to limit excessive inflammation during infection. Platelet-derived CD40L or other ligands can also modulate adaptive immunity. Additionally, both *in vitro* and *in vivo* evidence suggests that platelets also impact the development and functions of DCs to regulate T-cell and B-cell responses. According to previous reports, different products of platelets have different effects on DCs, thus changing their antigen presentation capacity.

All these reports indicated that in addition to hemostasis, platelets also play critical roles in the immune system, but the exact mechanism is still not clear. Given that platelet concentrates are widely used in clinical treatment and given the side effects of platelet transfusion, we need to consider the effects of platelets and their secreted molecules on immune cells, such as neutrophils, monocytes, B cells, T cells and DCs. Elucidating how platelets interact with these cells will contribute to broader application of platelet products and avoid adverse reactions.

## Author contributions

CY and HW mainly drafted and revised this review. XF and JH conceived and provided lots of advises about this review. FZ conducted the overall writing and previewed the draft and revised the manuscript. All authors contributed to the article and approved the submitted version.

## Funding

The present study was supported by grants Scientific Research and Development Fund of Kangda College, Nanjing Medical University (grant no. KD2019KYJJZD019) and the Jiangning District Social Undertakings Science and Technology Development Project (grant no. 2020SHSY0101).

## Conflict of interest

The authors declare that the research was conducted in the absence of any commercial or financial relationships that could be construed as a potential conflict of interest.

## Publisher’s note

All claims expressed in this article are solely those of the authors and do not necessarily represent those of their affiliated organizations, or those of the publisher, the editors and the reviewers. Any product that may be evaluated in this article, or claim that may be made by its manufacturer, is not guaranteed or endorsed by the publisher.
